# Characterization of macular lesions in punctate inner choroidopathy with spectral domain optical coherence tomography

**DOI:** 10.1007/s12348-011-0054-6

**Published:** 2012-01-01

**Authors:** Roomasa Channa, Mohamed Ibrahim, Yasir Sepah, Peykan Turkcuoglu, Jeong Hee Lee, Afsheen Khwaja, Elham Hatef, Millena Bittencourt, Jangwon Heo, Diana V. Do, Quan Dong Nguyen

**Affiliations:** Retinal Imaging Research and Reading Center (RIRRC), Wilmer Eye Institute, Johns Hopkins University, 600 North Wolfe Street, Maumenee 745, Baltimore, MD 21287 USA

**Keywords:** Choroid, Optical coherence tomography (OCT), Punctate inner choroidopathy (PIC), Retinal pigment epithelium (RPE)

## Abstract

**Purpose:**

Punctate inner choroidopathy (PIC) is an ocular inflammatory disease. Spectral domain optical coherence tomography (SD-OCT) allows detailed visualization of retinal and choroidal structures. We aimed to describe the retinal changes on SD-OCT associated with PIC lesions localized in the macula.

**Methods:**

Retrospective case series: PIC lesions not associated with choroidal neovascularization (CNV) and captured by macular SD-OCT scans were identified and characterized.

**Results:**

Twenty-seven PIC lesions from seven patients (eight eyes) were identified and classified into four categories according to disease activity and temporal changes. Among clinically inactive patients, two main patterns were noted on OCT: (1) retinal pigment epithelium (RPE) elevation with sub-RPE hyper-reflective signals and (2) localized disruption of outer retinal layers with choroid and Bruch's membrane (BM) generally spared. Clinically active patients demonstrated lesions with intact BM with RPE elevation that fluctuated with disease activity and sub-RPE hyper-reflective signals. Photoreceptor-associated bands on SD-OCT (PRs) were not visible during active disease, but returned to normal visibility when lesions were clinically stable. Seven lesions in patients without clinically detected activity demonstrated alteration of RPE elevation.

**Conclusion:**

SD-OCT can provide detailed structural characteristics of PIC lesions. RPE elevation is noted in many lesions while BM and choroid are spared. Photoreceptor-associated bands on SD-OCT appear compressed during clinically active stages and are visible during stabilization. OCT may provide information on activity not detected clinically.

**Electronic supplementary material:**

The online version of this article (doi:10.1007/s12348-011-0054-6) contains supplementary material, which is available to authorized users.

## Introduction

Punctate inner choroidopathy (PIC) is an ocular inflammatory disease mostly affecting young myopic women. It was described by Watzke et al. in 1984; diagnosis was based on the visualization of yellowish white punctate lesions on funduscopy apparently in the absence of ocular inflammation [1]. These lesions appeared to be located at the level of the choroid and retinal pigment epithelium (RPE). Imaging studies have revealed leakage of active lesions on fluorescein angiography (FA) [1] and evidence of involvement of choriocapillaris on indocyanine green angiography (ICG) [2, 3]. Autofluorescence of PIC lesions in correlation with activity has been described [4]. However, to date, the exact localization of origin of the lesions is not known.

In recent years, optical coherence tomography (OCT) has become a common non-invasive imaging modality for visualizing retinal structures in detail. The advances in OCT technology have given ophthalmologists a detailed morphological view of the retina, previously only possible from microscopic analysis of cadaveric eyes. Hassentein and Meyer, in a recent review, reported a high correlation between histological structure of the retina and the image acquired by spectral domain (SD)-OCT [5]. Other studies have demonstrated the usefulness of SD-OCT in visualization of microscopic structures of the retina, like disruption of the photoreceptors inner/outer segment junctions (IS/OS) in cases of chloroquine toxicity [6], serpiginous choroiditis [7], and acute multiple evanescent white dot syndrome [8], and disruption that was followed by recovery of the IS/OS in some cases of acute zonal occult outer retinopathy (AZOOR) [9].

We sought to utilize the enhanced imaging capability of SD-OCT to describe the retinal changes of PIC lesions.

## Materials and methods

Medical records of patients with a diagnosis of PIC, who were being followed in the outpatient clinic at the Wilmer Eye Institute between March 2008 and March 2010, by the uveitis specialist (QDN), were reviewed. The diagnosis of PIC was made clinically by one of the authors (QDN), based on funduscopic appearance of multiple punctate chorioretinal lesions localized mainly in the posterior pole and in the absence of iridocyclitis and vitreal inflammation. Institutional Review Board (IRB)/Ethics Committee approval was obtained, and HIPAA guidelines were followed for the study.

PIC patients enrolled in the study were on immunomodulatory therapy (IMT) and were evaluated in the clinics approximately every 2 months. Clinic visits during which the patients had SD-OCT measurements were included in the index study. All ophthalmic examination, fundus photographs, and FAs of each patient for those particular visits were reviewed to identify all PIC lesions for possible inclusion into the study. The patients were then classified according to clinical activity of the disease as *clinically active* or *clinically stable*. The criteria for development of clinical activity were eye-specific as a patient may have active disease in one eye and not in the other. Clinical activity was identified if a patient met one or more of the following criteria at any visit:

- Visual disturbances/changes noticed by the patient that are consistent with symptoms of previous attacks
- Indistinct borders of lesions with surrounding edema on contact lens biomicroscopy
- Hyperfluorescence of the lesion with blurred borders on FA (Fig. 2b).

Patients who did not meet any of the above criteria were considered to have clinically stable disease. SD-OCT scans from selected patient were then reviewed. Scans of lesions within the area of retina covered by the macular raster (central 20 × 15° of the posterior pole) were included in the study. Lesions that were immediately adjacent to choroidal neovascularization (CNV) scars, with distorted surrounding retinal layers, were excluded.

The selected lesions were reviewed systematically in the Retinal Imaging Research and Reading Center (RIRRC) at Wilmer to identify and characterize the abnormalities, which included the presence of abnormal signals, distortion of the normal retinal architecture, and loss or disruption of individual retinal layers. The characterized abnormalities were then classified according to the disease activity.

SD-OCT findings of all patients were studied on the first visit available in our records; this visit was designated BL. SD-OCT scans were then reviewed for all available visits after the BL visit. These visits were labeled according to their temporal relationship to BL. For example, a visit that occurred 1 month after BL was labeled “M1.” After all observations have been analyzed, we describe the SD-OCT characteristics of the lesions in clinically active and clinically stable patients including the temporal changes observed on SD-OCT in both categories.

The OCT scans from our study patients were captured using Spectralis HRA + OCT™ (Heidelberg Engineering Inc., Vista, CA, USA), which provides an axial resolution of 3.9 μm and a transverse resolution of 14 μm in tissue. It can acquire up to 100 B-scans at a particular location, allowing reduction of noise artifact [10]. Eye tracking option helps to avoid motion artifacts and allows registration of the data points, tracking them in all subsequent scans using a specific scan as a reference. The eye tracking allows pixel-to-pixel comparison of individual sections, which helps in tracking and following up changes in individual lesions over time [11].

## Results

Twelve eyes of nine patients met the clinical diagnosis of PIC. Two eyes were excluded because of extensive scarring secondary to CNV. Another two eyes were excluded because none of the PIC lesions fell within the area covered by the macular raster of the SD-OCT. Twenty-seven PIC lesions were identified in eight eyes of seven patients (six women, one man). Six patients had scans available for multiple visits, with length of follow-up ranging from 8 to 23 months; one patient was scanned only once. Ages ranged between 23 and 57 years with a median of 30 years (Table 1).

**Table 1:** Demographic and clinical characteristics of the seven subjects in the study

Patient	Age/gender	Clinical activity	Total number of lesions studied on SD-OCT	Number of lesions with SD-OCT changes over time (f/u period with SD-OCT scans available)	Clinical characteristics
1	57/f	No	2	0 (8 months)	Remained stable during f/u
2	39/f	No	6	6 (19 months)	Remained stable during f/u
3	26/m	Yes	6	3 (21 months)	OD: worsening of a pre-existing scotoma, VA decreased from 20/25 at the previous visit to 20/63; contact lens biomicroscopy showed increased blurriness of borders, hyperfluorescence with blurred borders on FA
					OS: blurred vision, yellow chorioretinal lesion superior to the fovea, hyperfluorescence with blurred borders on FA
4	24/f	No	3	0 (6 months)	Remained stable during f/u
5	30/f	No	3	1 (16 months)	Remained stable during f/u
6	46/f	No	5	No f/u available	No f/u available
7	23/f	Yes	2	2 (23 months)	OD: increased blurriness of vision; hyperfluorescence with blurred borders on FA

*f/u* follow-up, *SD-OCT* spectral domain optical coherence tomography, *VA* visual acuity, *FA* fluorescein angiography, *m* male, *f* female, *OD* right eye, *OS* left eye

At their first visit to our clinic, all seven patients had a history of CNV in at least one eye, had experienced recurrent disease activity in the past, and were on maintenance IMT during the entire period in which the index study was conducted.

**Fig. 1:**
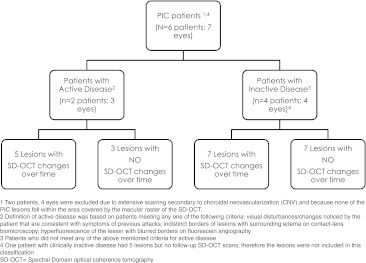
Categorization of PIC lesions in patients with active and inactive disease

Two patients were identified as having clinically active disease and five had clinically stable disease. Twenty-four of the 27 lesions included in the study showed involvement of the RPE layer on SD-OCT. Based on clinical activity of the disease and temporal changes of lesions on SD-OCT, we classified the 27 PIC lesions into four categories: (1) clinically active patients with lesions that showed temporal SD-OCT changes (five lesions from two patients), (2) clinically active patients with lesions that showed no temporal changes on SD-OCT (three lesions from one patient), (3) clinically stable patients with temporal changes on SD-OCT (seven lesions from two patients), and (4) clinically stable patients with no temporal changes on SD-OCT (seven lesions from three patients). Additional five lesions were identified in a patient with clinically stable disease but were not categorized as above because there were no follow-up SD-OCT scans available. Figure 1 shows categorization of the lesions.

## Lesions in clinically active patients

### Clinically active patients with lesions that showed temporal SD-OCT changes

#### Description of lesions at the time of clinical activity

Among a total of five lesions that were included in this category, RPE elevation with hyper-reflective sub-RPE signals was seen in four lesions. Bruch's membrane (BM) appeared to be intact in three lesions, disrupted in one, and not visible in one lesion. PRs were not visible in three lesions and partly visible in two lesions.

#### Temporal changes on SD-OCT

##### Patient 3 (three lesions)

Lesion 13: Lesion 13 was observed in the right eye of Patient 3 and demonstrated on the SD-OCT scan at BL as a small irregularity in RPE. It was observed to increase progressively in size over the course of 18 months prior to the detection of clinical activity.

**Fig. 2:**
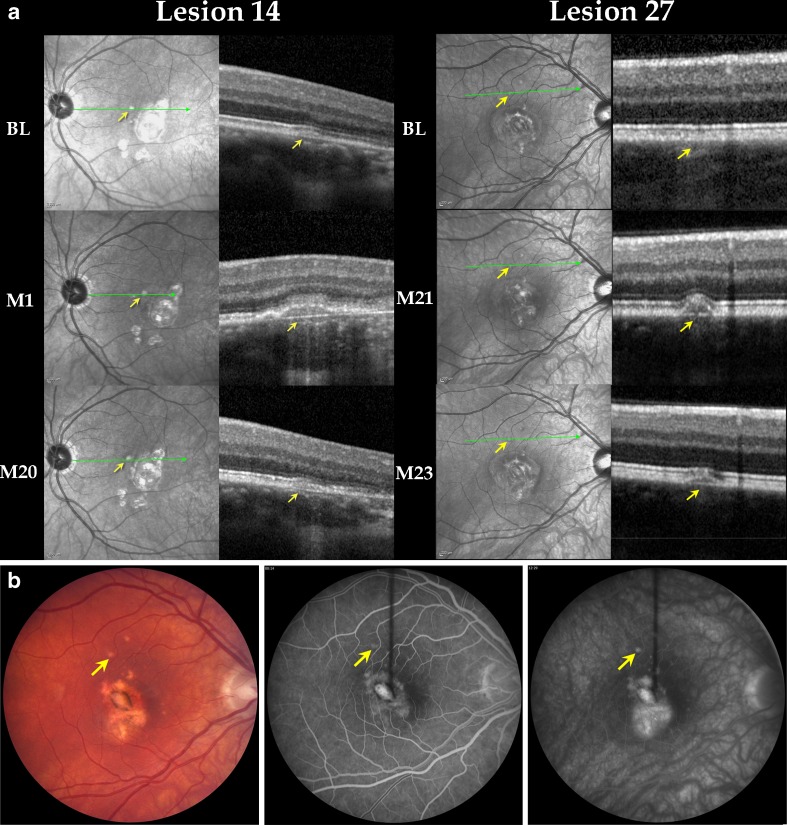
**a** Lesions in clinically active patients with temporal changes on spectral domain optical coherence tomography is a composite of IR and OCT images. The *arrows* point towards the lesion. The *first row* shows IR and OCT images at BL, the *middle row* shows IR and OCT images at the time of clinical activity, and the *bottom row* shows IR and OCT images of the same lesion at the last follow-up visit after resolution of clinical activity. *Left* (lesion 14): lesion (in the left eye) was initially seen as a small RPE elevation. Patient was clinically active at M1; during active disease, PRs were not visible. Resolution of RPE elevation and reappearance PRs were seen at M20. *Right* (lesion 27): lesion (in the right eye) was first seen at the time of clinical activity (M21) as a small RPE elevation, and PRs were partially visible. Two months later, the RPE elevation had decreased. *BL* first visit for which SD-OCT was available, *M* month, *PR* photoreceptor-associated bands on SD-OCT. **b** Color fundus photograph (*left*), early FA (*center*), and late FA (*right*) from Patient 7. The lesion marked by the *arrows* corresponds to lesion 27 as it appeared on color fundus and FA at M21 during clinical activity. The late FA shows mild leakage with blurring of the borders of the lesion. *FA* fluorescein angiogram, *M* month, *IR* infrared

Lesion 14: Review of SD-OCT scans of the left eye, acquired at the BL visit (i.e., 1 month prior to development of clinical activity), revealed that the patient had a lesion with RPE elevation and displaced PRs. At the time of clinical activity, RPE elevation had increased, and PRs were not visible. Over the course of the next 19 months, RPE elevation decreased, and PRs became visible again (Fig. 2: lesion 14).

##### Patient 2 (two lesions)

Lesions 26 and 27: Both lesions showed RPE elevation at the time of clinical activity associated with displacement of PR, followed 2 months later by resolution of RPE elevation and clear visibility of PR (Fig. 2: lesion 27).

### Clinically active patient with lesions that had no temporal changes on SD-OCT

#### Patient 3 (three lesions)

The patient was judged based on the above-mentioned criteria to have clinically active PIC disease. This category describes the characteristics of three lesions, in Patient 3, which remained unchanged over 21 months of follow-up. On SD-OCT scans, they appeared as localized fibrotic lesions with RPE and PR disruption, accompanied by outward pulling of outer nuclear layer (ONL) and outer plexiform layer (OPL).

## Lesions in clinically stable patients

Among the clinically stable patients, two main patterns were noted on SD-OCT: (1) RPE elevation with sub-RPE hyper-reflective signals and (2) no RPE elevation, but there was localized disruption of outer retinal layers including the ONL, OPL, PR, and RPE layers, with sparing of choroid and BM.

### Clinically stable patients with lesions that showed temporal changes on SD-OCT

#### Patient 2 (six lesions)

**Fig. 3:**
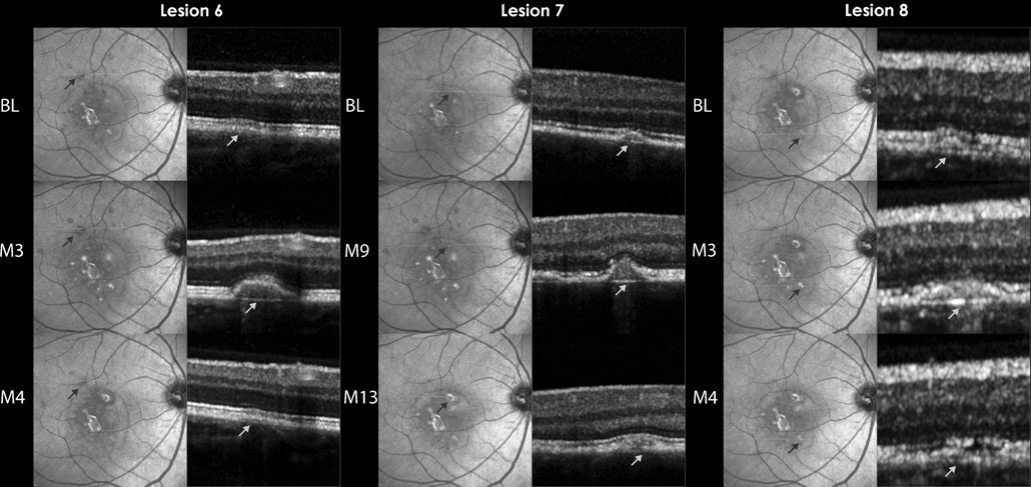
Lesions in clinically stable patient with temporal changes on spectral domain optical coherence tomography is a composite of IR and OCT images showing three lesions from the right eye of Patient 2. All three lesions (6, 7, and 8) showed retinal pigment epithelium (RPE) elevation with disappearance of PRs, followed by resolution of the RPE elevation and reappearance of PRs. *BL* time point when lesion was first observed on SD-OCT, *M* month, *PRs* photoreceptor-associated bands on SD-OCT, *IR* infrared

All six lesions showed temporal changes on SD-OCT, which were not associated with any evidence of clinical activity. The changes included alteration in the extent of RPE elevation and sub-RPE signals. In all six lesions, it was observed that the *visibility* of the PRs was related to the extent of RPE elevation; non-visibility of PRs was associated with progression of the RPE elevation, and the visibility was associated with the regression and resolution of the RPE elevation. BM was intact in all six lesions (Fig. 3: lesions 6, 7, and 8).

#### Patient 5 (one lesion)

A localized area of RPE elevation, with intact BM and intact PRs. RPE elevation had resolved on a follow-up scan 1 year later.

### Clinically stable patients with lesions that showed no temporal changes on SD-OCT

#### Patient 1 (two lesions)

**Fig. 4:**
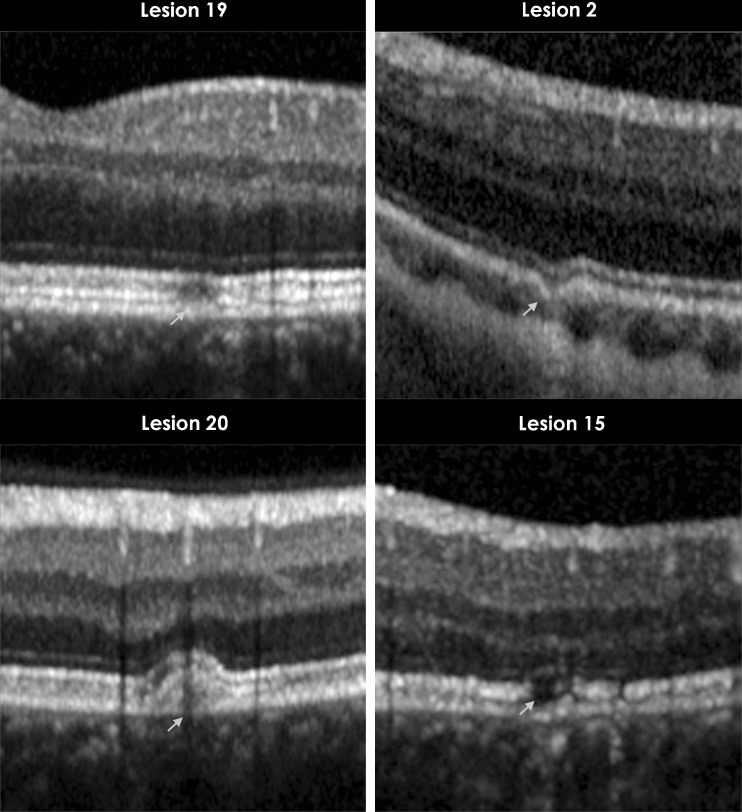
Clinically stable lesions that have remained unchanged on spectral domain optical coherence tomography scans during multiple visits. This figure shows magnified SD-OCT scans. The *arrows* point to the lesions. *Top left* (lesion 19): focal retinal pigment epithelium (RPE) disruption remained unchanged over a period of 16 months. *Top right* (lesion 2): focal irregularity of RPE and photoreceptor IS/OS junctions with choroidal thinning, unchanged over a period of 8 months. *Bottom left* (lesion 20): RPE elevation with sub-RPE signal, unchanged over a period of 16 months. *Bottom right* (lesion 15): focal disruption of photoreceptor IS/OS junctions with thinning of the ONL, unchanged over a period of 6 months. Repeated OCT scans at subsequent visits did not change in appearance compared to the images illustrated in these figures, and therefore, are not shown. *SD-OCT* spectral domain optical coherence tomography, *RPE* retinal pigment epithelium, *IS/OS* inner segment/outer segment, *ONL* outer nuclear layer, *mos* months

Lesions remained unchanged on SD-OCT over a follow-up period of 8 months. A funduscopically visible curvilinear lesion was observed on SD-OCT as having a localized hyper-reflective sub-RPE signal with back shadowing; BM appeared to be intact. In the same eye, further towards the inferior arcade, there was another lesion with focal disruption of the RPE; the lesion seemed to be pulled (dragged) outwards with back shadowing, and there was an associated focal choroidal thinning (Fig. 4: lesion 2).

#### Patient 4 (three lesions)

Lesions remained unchanged on SD-OCT over a period of 6 months. All three lesions were morphologically similar to each other and revealed focal disruption of PRs (Fig. 4: lesion 15).

#### Patient 5 (two lesions)

Both lesions remained unchanged on SD-OCT over a period of 16 months of follow-up. On SD-OCT, lesion 19 was described as a focal RPE disruption and lesion 20 as a stable RPE elevation with sub-RPE signals.

## Discussion

In this index study, we aimed to characterize the various lesions seen clinically at different stages in PIC based on findings visualized on SD-OCT. The eye tracking capability of the Spectralis® SD-OCT has allowed for exact (in location) comparisons of lesions over time. The majority of lesions (24 of a total of 27) in our study, irrespective of clinical activity, showed involvement of the RPE. RPE changes included RPE elevation with sub-RPE signals and RPE discontinuity. In the first description of PIC lesions, Watzke et al. described serous retinal detachments occurring over choroidal lesions [1]. In our review of 27 lesions, the most commonly observed morphology of lesion on SD-OCT involved RPE elevation, with sub-RPE signals localized over an apparently intact BM. Furthermore, Watzke et al. described that serous RPE detachments occurred either at the first appearance of a new lesion or during recurrence [1]. Our findings are consistent with the clinical observations made by Watzke: clinically active patients in our study had lesions that demonstrated RPE elevation with sub-RPE signals.

Temporal SD-OCT changes were observed in all clinically active patients and also in seven lesions of two other patients who were not identified to be clinically active. Temporal SD-OCT changes in four of the five lesions in clinically active patients were very similar to the changes observed in seven lesions in the two clinically stable patients. There could be several explanations for such observation. One possible explanation is that the high-resolution property of SD-OCT makes it a sensitive imaging modality which may identify changes before they become clinically detectable. Such hypothesis is further supported by the fact that SD-OCT changes in three lesions of Patient 3 were visible up to 21 months before identification of clinical activity.

PIC is usually a benign disease but has the potential to cause severe visual loss if complicated by CNV and sub-retinal fibrosis: rates of up to 69% and 56%, respectively, were reported in our study of 77 PIC patients, and 75% of patients in a series of 12 patients had CNV [12, 13]. Essex et al. studied clinical outcomes of 271 eyes with typical and atypical PIC. Typical PIC included eyes with small lesions (less than or equal to a quarter of a disc diameter) located within the arcades, while atypical PIC included eyes with larger lesions located within and outside the arcades. No significant differences in demographics and clinical outcomes were identified between the two groups. They reported that presence of PIC lesions in fellow eyes of patients with unilateral CNV was a risk factor for development of CNV in the fellow eyes as well [14], hence emphasizing the importance of immediately identifying and treating any recurrence in disease activity. Previously, we have shown that the employment of IMT such as mycophenolate mofetil may help to decrease the frequency of attacks in recurrent PIC disease [4]. Our current results indicate that SD-OCT may play a role in early identification of recurrence of disease activity and development of new lesions.

An interesting phenomenon was noted while we were analyzing the SD-OCT scans. Patients 2, 3, and 7 had several lesions with RPE elevation and non-visibility of the overlying PRs. Review of latter scans of the same lesions revealed that the RPE elevation gradually resolved, and the PRs became visible. A possible explanation for this phenomenon may be that in the initial stages, there is elevation of RPE and compression of PRs. Resolution of the RPE elevation restored the PRs to their original position. The non-visibility of PRs persisted during follow-up in three lesions of Patient 5. Spaide et al. demonstrated loss of PRs in AZOOR complex of diseases [9]. They described that the recovery of PRs on SD-OCT was linked to the preservation of the ONL. Such prediction is possible in cases of isolated loss of PR as seen in Patient 5. However, in lesions with RPE elevation, visualization of the ONL and prediction of PR recovery based on its appearance is quite impossible.

In our scans, we were unable to identify changes in the choroid in all except one lesion. It was surprising because the first report from Watzke mentioned choroidal involvement of lesions, and eyes with PIC have been shown to have involvement of choriocapillaris on ICG [1–3]. The lack of choroidal involvement raised questions regarding the origin of the pathologic process in PIC: does the process begin in the choroid or does it involve the choroid in later stages? In Patient 3, lesion 9 began with an intact BM, but in later scans, the BM appeared to be disrupted, suggesting that in the initial stages, the pathological process was localized above the choroid, and only after disruption of the BM, it progressed into the choroid. However, there are limitations to such finding in our study. It has been reported that in order to optimally visualize the choroid, enhanced depth imaging (EDI) technique should be employed [15]. Our scans were intended to optimally view the retinal layers; therefore, we cannot make a definite conclusion about the extent of choroidal involvement based on OCT scans. Also, our study was a retrospective review, and it is difficult to draw definitive conclusions about the disease process.

The Japanese literature has previously reported TD-OCT findings of four patients with PIC disease [16, 17]. The investigators reported the formation of what they described as a “hump” at the site of the active PIC lesion and changes in reflectivity of the RPE-BM and choriocapillaris complex as the lesion progressed over time. It is difficult to compare their findings to ours as TD-OCT, at best, provides minimal detail on morphology of individual retinal layers. Stepien and Carroll described SD-OCT changes in an eye with PIC disease. At the time of clinical activity, there was homogenous outer retinal thickening overlying chorioretinal lesions which resolved 3 months after treatment. Although the descriptive terms used by them are not the same as the terms employed in our report, the SD-OCT images presented by them during development and resolution of disease activity are strikingly similar to ours [18].

The definitive pathophysiology of white dot syndromes has been much debated throughout the years [19]. SD-OCT may provide further evidence to distinguish among these disorders. Spaide et al. described lesion characteristics of 13 eyes with multifocal choroiditis with panuveitis (MCP) on SD-OCT [9]. Although they demonstrated loss of PRs, none of the eyes demonstrated RPE elevation as seen in our study of PIC patients. Could such finding be further evidence that PIC and MFC are different disease entities [20]?

One of the hypotheses for the pathogenesis of PIC is that the disease is secondary to the development of myopic cracks in BM. However, PIC is mostly seen in patients with moderate myopia. The assessment of integrity of BM was limited in some lesions due to the abnormal reflectivity of light by adjacent structures. However, when clearly visible, it appeared to be intact in the majority of the lesions in our study. Review of SD-OCT scans of Patient 3 demonstrated destruction of the BM on later scans when compared to earlier scans.

SD-OCT may be able to provide detailed structural characteristics of PIC lesions and may serve as a useful, non-invasive means of identifying disease activity in patients with PIC. RPE elevation is noted in many lesions, while BM and choroid are spared. PRs appear to be compressed during RPE elevation and resume clear visibility upon resolution of the RPE elevation. Therefore, SD-OCT may provide information on disease activity that is not detected clinically or on FA. Further systematic, prospective SD-OCT studies of PIC lesions will most likely provide additional insights about the clinical course of PIC, and may provide clinicians with a non-invasive imaging tool in the management of patients with PIC.

## Electronic supplemental material

Below is the link to the electronic supplementary material. ESM 1 (PDF 22 kb)
